# *In vivo* measurement of hemodynamic information in stenosed rat blood vessels using X-ray PIV

**DOI:** 10.1038/srep37985

**Published:** 2016-11-28

**Authors:** Hanwook Park, Jun Hong Park, Sang Joon Lee

**Affiliations:** 1 Center for Biofluid and Biomimic Research, Department of Mechanical Engineering, Pohang University of Science and Technology (POSTECH), Pohang, 790-784, South Korea

## Abstract

Measurements of the hemodynamic information of blood flows, especially wall shear stress (WSS), in animal models with circulatory vascular diseases (CVDs) are important to understand the pathological mechanism of CVDs. In this study, X-ray particle image velocimetry (PIV) with high spatial resolution was applied to obtain velocity field information in stenosed blood vessels with high WSS. 3D clips fabricated with a 3D printer were applied to the abdominal aorta of a rat cadaver to induce artificial stenosis in the real blood vessel of an animal model. The velocity and WSS information of blood flows in the stenosed vessel were obtained and compared at various stenosis severities. *In vivo* measurement was also conducted by fastening a stenotic clip on a live rat model through surgical intervention to reduce the flow rate to match the limited temporal resolution of the present X-ray PIV system. Further improvement of the temporal resolution of the system might be able to provide *in vivo* measurements of hemodynamic information from animal disease models under physiological conditions. The present results would be helpful for understanding the relation between hemodynamic characteristics and the pathological mechanism in animal CVD models.

Hemodynamic parameters, such as blood flow velocity, WSS, and oscillatory shear index and pressure, are associated with the occurrence and progress of CVD^1^, including atherosclerosis, stroke, and ischemic heart disease. WSS is one of the most important parameters that affect the occurrence of atherosclerosis^2^. Given that WSS can be estimated with the velocity gradient of blood flow in the near-wall region, accurate measurements of the velocity field information of blood flows under *in vivo* conditions are important.

Many non-invasive measurement techniques, such as X-ray PIV^3^, magnetic resonance imaging^4^, and ultrasound PIV^5^, have been utilized to obtain blood flow hemodynamic information. Velocity fields with high spatial resolution are essential for the accurate estimation of WSS. Hence, synchrotron X-ray PIV with high spatial resolution was employed in this study to measure blood flow velocity field information and WSS in the blood vessels of a rat stenosis model. X-ray PIV has been utilized to investigate hemodynamic characteristics in blood vessels^6^,^7^, a stenosis model^8^, and a rat extracorporeal loop^9^. To obtain such hemodynamic information in blood vessels through X-ray PIV, fine particles, such as iopamidol encapsulated by polyvinyl alcohol^10^, gold nanoparticles^11^, ultrasound contrast agents^12^, and CO_2_ microbubbles^13^, are introduced as flow tracers. These tracer particles exhibit advantages and disadvantages depending on the X-ray beam and experimental conditions. In the present study, CO_2_ microbubbles were utilized as flow tracers to measure blood flows in stenosed vessels.

CO_2_ gas has long been used as a contrast agent in X-ray angiography^14^ because it has a low hypersensitivity reaction compared with iodine as a contrast agent^15^. However, because of the two-phase flow features of CO_2_ gas in blood, CO_2_ gas can induce a reverse flow when trapped in a blood vessel. To overcome this limitation, CO_2_ microbubbles are used as a contrast agent in X-ray imaging^16^ and sonographic angiography^17^,^18^. We used CO_2_ microbubbles as flow-tracing particles in X-ray PIV measurements and demonstrated their traceability under steady^13^ and pulsatile flow conditions^9^. Recently, we obtained velocity field information in a rat extracorporeal loop^9^ by using CO_2_ microbubbles and validated the *in vivo* feasibilities of using such microbubbles^7^.

A disturbed flow in blood vessels affects the vascular endothelium^19^. Among the various configurations that cause disturbed flows in the circulatory vascular system, the flow characteristics in stenosed vessels have been widely investigated^20^,^21^,^22^. A number of simulation and experimental studies have been conducted to investigate hemodynamic and pathological characteristics in stenosed vessels^20^,^23^. In particular, *rodent* stenosis models have been widely utilized to reveal the relationship between hemodynamic characteristics and pathological features^24^,^25^,^26^ because stenosed vessels are easily induced by surgical treatment^27^,^28^ and/or attachment of stenosis devices^29^,^30^. With such rodent models, the roles of WSS were studied in terms of gene expression, protein level, and pathological properties. However, direct comparisons of hemodynamic and pathological characteristics are difficult because of technological limitations in the accurate measurement of blood flow velocity field information s. Hence, computational simulations have been utilized as an alternative to estimate WSS in stenosed vessels. However, precise estimation of WSS in stenosed blood vessels is difficult because of the non-Newtonian fluid characteristics of blood caused by the presence of red blood cells. Moreover, simple and easy fabrication of a stenosis is another limiting factor in the analysis of the effect of various stenosis shapes on the variation in WSS.

To overcome these technical problems, we investigated the hemodynamic information of blood flows in simple stenosed blood vessels by utilizing X-ray PIV velocity field measurement technique. First, a 3D printer was used to fabricate 3D stenosis clips with various severities to artificially induce stenotic blood vessels with a predetermined configuration in a rat model. Second, velocity field information and WSS distributions in the stenotic blood vessels were measured through X-ray PIV. Finally, the stenosis clips were applied to the rat model to demonstrate *in vivo* measurement of blood flows in animal models with diseases. The results can be used to investigate the effect of geometric parameters of stenosed blood vessels on the hemodynamic features of animal models with CVDs.

## Results

**Figure 1:**
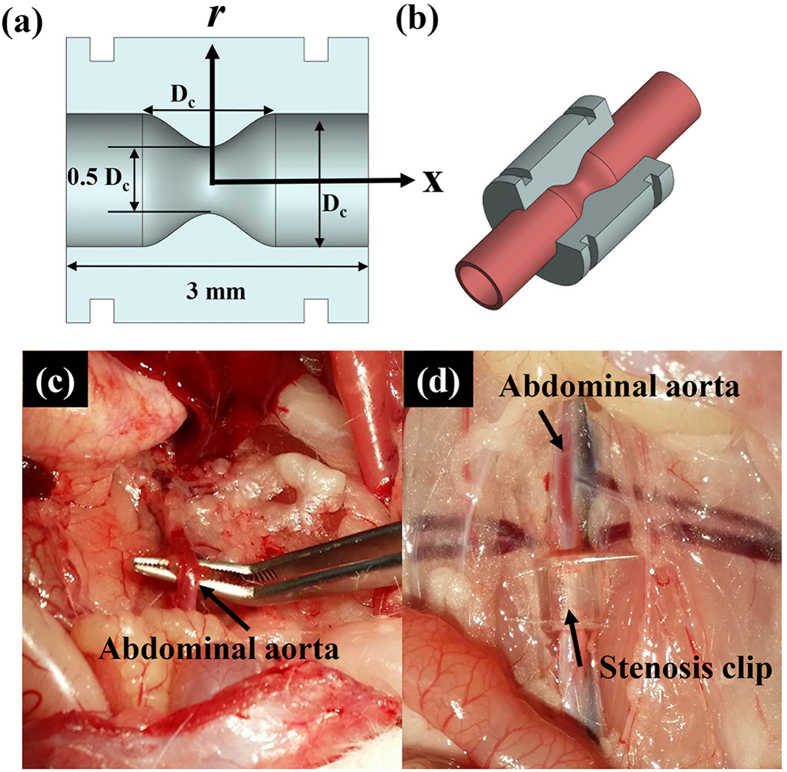
(**a**) Geometric parameters of a concentric stenosis clip with severity of S_c_ = 50%. (**b**) Illustration of a 3D stenosis clip installed in a straight blood vessel. (**c**) Abdominal aorta in a Sprague–Dawley rat. (**d**) Abdominal aorta attached with the stenosis clip.

**Figure 2:**
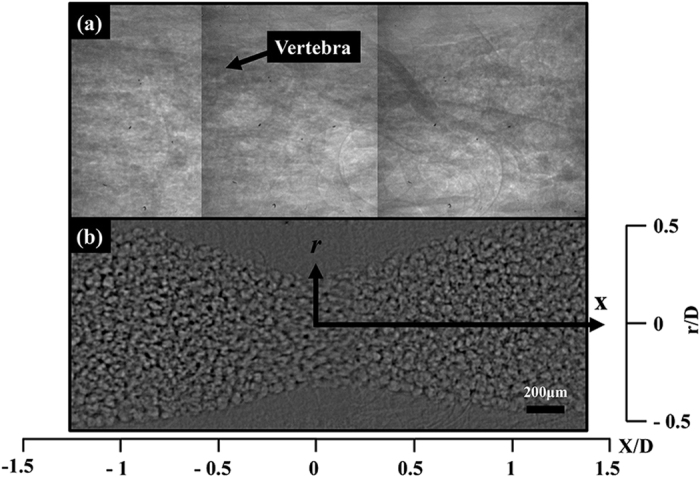
Typical X-ray image of blood flow in the stenosed vessel of S = 36% (**a**) before and (**b**) after digital image processing.

**Figure 3:**
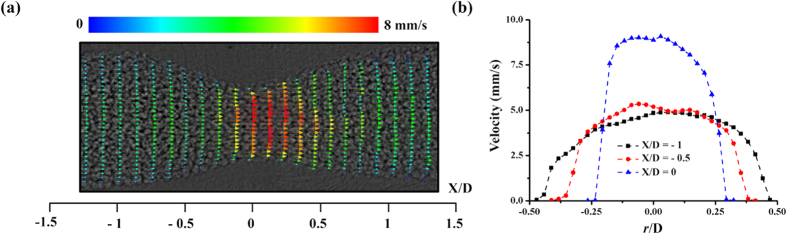
(**a**) Instanteneous velocity field in the stenosed blood vessel of S = 36%. (**b**) Comparison of mean velocity profiles in three upstream locations.

Figure 1a shows the geometric parameters of a 50% concentric stenosis clip used in this study. S_c_ and S denote the severity of the stenosis clip and real blood vessel, respectively. Figure 1b shows the 270° part of the stenosis clip attached to a straight blood vessel of a rodent model. The material of the stenosis clip was VisiJet M3 crystal. A groove was engraved at both ends of each clip for tightening. Figure 1c and d show the abdominal aorta before and after clip installation, respectively. Figure 2a shows raw X-ray images of blood flow in the stenosed vessels. Three adjacent X-ray images were combined in consideration of the streamed blood flow. To remove the unnecessary information of surrounding tissues and the effects of inhomogeneous X-ray beam fluctuations, the flat field correction method was applied^31^. A spatial frequency filter was used to remove noises caused by image intensification procedures^32^. The effects of digital image processing techniques on the accuracy of X-ray PIV measurement are well described in our previous work^7^. Figure 2b shows a typical X-ray image of the blood flow in the stenosed vessels to which various image processing techniques were applied. In the figure, *D* stands for the diameter of the straight abdominal aortic vessel before the clip was attached. *D* is smaller than *D*_*c*_ because of the presence of vessel wall thickness. Table 1 summarizes the geometrical parameters of the three stenosis clips tested in this study. *L* and *r*_o_ are the length of the stenosis and radius of the stenosis throat, respectively. Figure 3a presents the instantaneous velocity field of blood flow in the rat stenosis model with S = 36%. The maximum velocity is approximately 9.2 mm/s. The mean velocity profiles at three X/D positions were compared, and the results are shown in Fig. 3b. The mean velocity profile was obtained through ensemble averaging of 400 instantaneous velocity fields.

**Figure 4:**
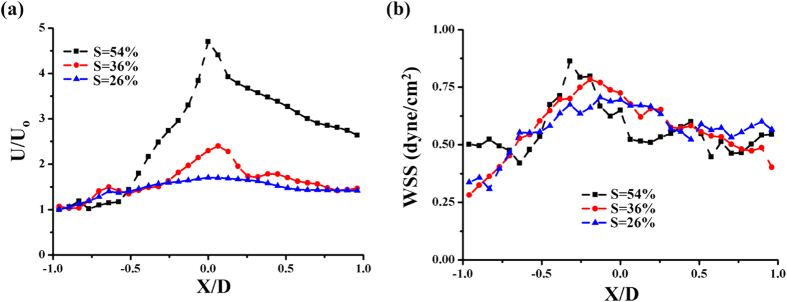
Variations in (**a**) normalized velocity and (**b**) WSS in accordance with stenosis severity S.

The mean flow velocity and WSS information were obtained by fastening a 3D stenosis clip with three different stenosis severities to a rat cadaver model. Figure 4a shows the variations in centerline velocity in the stenosis model in accordance with stenosis severity (S). The velocity values were normalized by inlet velocity (U_0_). In each severity condition, the maximum velocity approximately occurred at a location slightly away from the crest of the stenosis. The normalized maximum velocity (U/U_0_) was 4.73, 2.38, and 1.71 for stenosis severity of S = 54%, 36%, and 26%, respectively. Figure 4b shows the variations in WSS in the axial direction in accordance with stenosis severity. The peak values of WSS were 0.86 dyne/cm^2^ (X/D = −0.32), 0.79 dyne/cm^2^ (X/D = −0.192), and 0.71 dyne/cm^2^ (X/D = −0.128) for stenosis severity S = 54%, 36%, and 26%, respectively.

**Figure 5:**
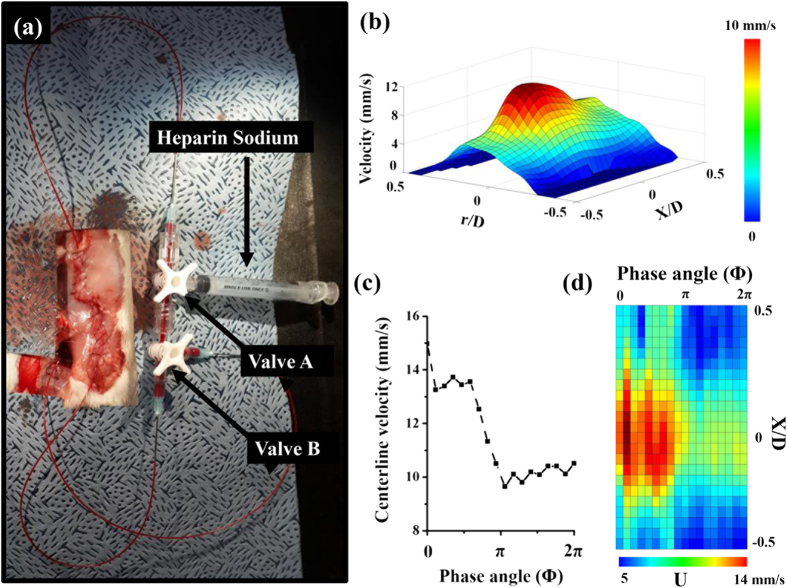
(**a**) Photograph of a rat stenosis model for *in vivo* measurement of blood flow. (**b**) Variation in radial velocity profile in the stenosed blood vessel at a phase angle of Φ = 0.44 π. (**c**) Phasic variation of centerline velocity. (**d**) Variation in centerline velocity of the stenosed blood vessel in accordance with phase angle Φ.

The hemodynamic information of blood flow in a live rat stenosis model was measured under *in vivo* conditions through simple surgical intervention to reduce the flow rate passing through the stenosed vessel. Figure 5b shows a typical velocity distribution of blood flow in the stenosed vessel of a rat model at Φ = 0.44 π. The corresponding peak velocity was approximately 13.72 mm/s. One pulsating period was divided into 18 phase angles from the systole phase (Φ = 0) to the diastole phase (Φ = 2 π). The velocity profiles were obtained through ensemble averaging of 20 instantaneous velocity fields at each phase. Figure 5c presents the phasic variation of the maximum centerline velocity. The maximum and minimum centerline velocities were 14.97 and 9.64 mm/s, respectively. The pulsatile index (PI) was about 0.43, and the heart rate was approximately 1.35 bpm. Figure 5d shows the variation in centerline velocity along the stenosed vessel in accordance with the phase angle. The peak centerline velocities occurred at approximately X/D = 0 for all phase angles. In addition, the length of the jet-like flow in the post-stenotic region decreased as the phase moved from systole to diastole.

## Discussion

Understanding the hemodynamic features of blood flows during stenosis is important such blood flows have a strong influence on the further progression of lesions, fractures, and ruptures^20^. Among the hemodynamic factors involved in CVDs, WSS is one of the important ones that affect the occurrence and progression of atherosclerosis^2^. WSS in a stenosed blood vessel also affects platelet activation and aggregation, which lead to cardiovascular diseases^33^,^34^. Recent reports have shown that both low and high shear stresses affect the formation of atherosclerosis in an animal model^35^. Therefore, an advanced blood flow measurement technique with high spatial resolution is required to investigate hemodynamic information in stenosis vessels accurately and systematically because WSS is evaluated by calculating the velocity gradient in the region near the vessel wall. Moreover, the hemodynamic characteristics of blood flow in a stenosis model should be measured with high accuracy to reveal the pathological mechanism of cardiovascular diseases because WSS directly induces variations in mRNA and protein expression^30^. Therefore, an advanced X-ray PIV technique with high spatial resolution was employed in this study to obtain the velocity field information of blood flows in stenosed blood vessels accurately.

Although *in vivo* experiments on hemodynamic phenomena in a stenosis model are essential, preparing animal models with stenosed blood vessels is difficult. Rats have been widely utilized as animal models of CVDs because they are relatively cheap and their blood vessels are easy to handle because of the small vessel size^27^. Therefore, a stenosed blood vessel can be easily prepared by attaching a clip fabricated with a 3D printer to a rat model. This method has several advantages, such as easy configuration of various stenosis, simple fabrication, and low cost. In this study, we used a rat model to measure the blood flow in a stenosed blood vessel. The main objective of this study is to demonstrate the direct measurement of blood flow in a stenosed blood vessel arbitrarily formed in a rodent model. This type of experimental model is appropriate to obtain hemodynamic information of blood flows in stenosis models under realistic physiological conditions.

The statistical average of difference between severity of blood vessel S and severity of clip S_c_ is 10.4 ± 2.7%. In this study, 3D clips were fabricated using a 3D printer and each of them was applied to the abdominal aorta of a rat model to induce artificial stenosis by utilizing elastic deformation basically. Therefore, many parameters are determined by the elastic deformation of blood vessels. Nonlinear and anisotropic behaviors of blood vessels are also reflected in the shape of stenosis model^36^. The blood pressure in the blood vessels is also influenced by the deformation of blood vessels. Moreover, it is essential to minimize clearance between the 3D clip and blood vessel of each rodent model. Therefore, the mechanical properties of blood vessels and accurate measurements of blood pressure and geometric parameters with high precision are important to achieve better shape fidelity of the 3D stenosis clip model.

The velocity and WSS information of blood flow in a rat cadaver model were measured through X-ray PIV. The rat cadaver model was utilized to compare the variations in hemodynamic information without considering physiological differences. The normalized centerline velocities increased with increasing stenosis severity S. The acquisition of accurate velocity information in the near-wall region is important in calculating WSS values. Conventional PIV analysis has a technical limitation in the accurate measurement of WSS on a curved wall because of the absence of particle information in consecutive flow images. Therefore, in this study, the interrogation windows located near the vessel walls were rotated to search for the exact velocity gradient in the near-wall regions. Figure 4b shows a comparison of WSS distributions along the stenosed vessel wall in accordance with stenosis severity. The peak values of WSS occurred at locations prior to the throat of the stenosis (X/D < 0) in all cases. The peak location shifted forward to the throat, and the corresponding WSS peak value increased with the increase in stenosis severity. This result is consistent with the result of a previous study on WSS in a stenosed vessel^8^. Although recirculation flow was not clearly observed in this study because of the flow with a low Reynolds number, the recovery length of WSS is longer in the downstream than in the upstream of the stenosis.

To demonstrate the feasibility of *in vivo* measurement of real blood flows in stenosed vessels, a 3D stenosis clip was applied to a live rat. The 3D stenosis clip was fastened to a straight blood vessel of a live rat; a flow-rate control system was included to reduce the flow rate and match the tolerance limit of the X-ray PIV system. The velocity information in the stenosed vessel of the live rat was compared according to the phase angle. Although the use of this stenosis clip is an innovative procedure in experimental studies on CVD models, technological improvements are required to acquire hemodynamic information with high accuracy in real physiological conditions. X-ray images contain all flow information along the X-ray beam propagation. Therefore, extractions of 2D velocity profiles from the amassed velocity information of Newtonian fluid flow and blood flows have been demonstrated in previous studies^3^,^13^. Decomposition of the smearing effects of X-ray images is another method to extract 2D velocity profiles. However, current algorithms are insufficient to reveal 2D velocity information of blood flows in a stenosis model. Given that the flow characteristics in the stenosis region are different from those in a circular straight pipe, the z-directional velocity components (along the X-ray beam propagation) are influenced by the amassed information. Hence, further studies are required to extract 2D velocity profiles from 3D volumetric velocity information of unsteady pulsatile blood flows in an axisymmetric conduit. In addition, the temporal resolution should be improved. We reduced the flow rate by bypassing some parts of blood flow in the *in vivo* measurement of real blood flows because the temporal resolution was insufficient to directly measure real blood flows in the abdominal aorta^27^. Therefore, the measured hemodynamic information, such as PI and flow velocity, is somewhat different from that under real physiological conditions. These technical limitations can be overcome with advances in the X-ray source or sensitivity of recording devices^6^,^7^. Polychromic X-ray beam can also be used to increase the temporal resolution of the X-ray PIV system. X-ray tomographic PIV^12^ and holographic X-ray imaging techniques^37^ were introduced as alternative means to measure 3D volumetric flow information. Although several technical limitations remain, the present measurement technique has a strong potential for the simultaneous measurement of hemodynamic information and pathological characteristics in a stenosis animal model.

## Conclusion

In this study, hemodynamic information, including WSS of blood flows in the stenosed vessel of a rodent model, was obtained through X-ray PIV with high spatial resolution. 3D clips fabricated with a 3D printer were utilized to induce artificial stenosis in real blood vessels. The hemodynamic information of blood flows in three stenosed vessels with different severities was quantitatively compared. The feasibility of *in vivo* measurement was also demonstrated by measuring real pulsatile blood flow in a live rat. To obtain precise hemodynamic information from CVD animal models, technological improvements, especially in the temporal resolution of the X-ray PIV system, are required in the near future. The presented X-ray PIV technique has a strong potential for *in vivo* measurement of hemodynamic information in CVD animal models.

## Materials and Methods

### Stenosis model

Three stenosis clips with severities of S_c_ = 30%, 40%, and 50% were fabricated with a 3D printer (ProJet^®^ 3510 HDPlus, USA); the laminating thickness was 16 μm. The inner radius *r*(x) of the stenosis clip can be expressed by the equation^38^


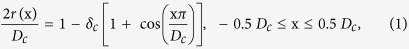


where *D*_*c*_ is the inner diameter of the non-stenosed normal part of the stenosis clip. The internal diameter of the straight part of the clip (*D*_*c*_ = 1.4 mm) was determined through preliminary experiments in consideration of the measured vessel diameter of the abdominal aorta. The vessel blockage ratio *δ*_*c*_ of this stenosis clip is 0.25, indicating a 50% reduction in vessel diameter. Each fabricated stenosis clip is composed of two constituent parts of 270° and 90°.

### Animal cadaver model

**Figure 6:**
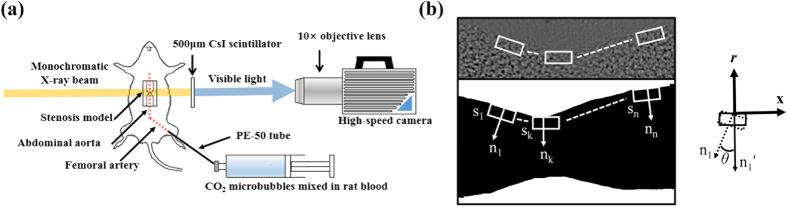
(**a**) Schematic of the experimental setup with a rat model with stenosed blood vessel. (**b**) Normal vectors and interrogation windows in the near-wall region.

Figure 6a shows a schematic of the experimental setup with a rat model. A male Sprague–Dawley rat (12 weeks old, 356 g) was anesthetized through intramuscular injection of ketamine (100 mg/kg) and xylazine (10 mg/kg). A PE-50 tube (ID = 0.58 mm, polyethylene tube) was cannulated to the right jugular vein to inject 500 IU/mL/kg heparin to prevent blood coagulation. The PE-50 tube was connected to the femoral artery to circulate blood in the rat model. To supply the mixture of blood and CO_2_ microbubbles, the PE-50 tube cannulated to the femoral artery was connected with a silicon tube (ID = 1.5 mm). A syringe pump (PHD 2000, Harvard Apparatus, USA) was utilized to supply the mixture at a flow rate of 0.5 mL/min. The clips were installed at the straight part of the abdominal aorta in the rat cadaver. All procedures performed on the test animals were approved by the Animal Care and Ethics Committee of POSTECH. The experiments were conducted in accordance with the approved guidelines.

### CO_2_ microbubbles

CO_2_ microbubbles were fabricated through mechanical agitation^39^,^40^. CO_2_ gas and 5% human serum albumin were mechanically agitated in a homogenizer (IKA-T25 Digital ULTRA-TURRAX, IKA, Germany) at 15,000 rpm for 7 min to generate CO_2_ microbubbles. The procedures to generate CO_2_ microbubbles are well described in our previous study^13^.

### *In vivo* animal model

Figure 5a shows an *in vivo* rat model with connecting loops for injection of CO_2_ microbubbles and heparin sodium. The flow rate control system utilized in this study is also shown in Fig. 5a. Supplementary Fig. S1 illustrates the surgical procedures applied to establish the *in vivo* rat stenosis model. The abdomen of the rat was cut open, and the abdominal aorta was removed (Fig. S1a). Two clamps were used to block the blood vessels in the celiac (Fig. S1b) and caudal arteries (Fig. S1c). A PE-50 tube was cannulated to the upper part of the genital artery to extract blood (Fig. S1d). The opposite end of the PE-50 tube connected to valve A (in Fig. 5a) was inserted to the lower part of the genital artery to supply a mixture of blood and CO_2_ microbubbles (Fig. S1e). Subsequently, the two clamps were removed, and the stenosis clip with a severity of S_c_ = 30% was fastened to a straight part of the abdominal aorta (Fig. S1f). The PE-50 tube linked with valve B was connected to the femoral vein (Fig. S1g). Blood was divided with valve B (Fig. 5a) to reduce the flow rate and streaming velocity. Heparin sodium was supplied through valve A, which was also used to supply the mixture of CO_2_ microbubbles and blood. The skin of the rat model was sutured to prevent external exposure of its internal organs (Fig. S1h). A syringe pump (PHD 2000, Harvard Apparatus, USA) was utilized to circulate the mixture of blood and CO_2_ microbubbles at a flow rate of 0.1 mL/min.

### X-ray PIV experiment

The X-ray experiment was conducted with the 6 C biomedical imaging beamline (6 C BMI) of Pohang Light Source (PLS-II). The beam current and storage energy of PLS-II were 360 mA and 3 G eV, respectively. A monochromatic X-ray beam used in this study and the corresponding beam flux was 1.2 × 10^12^ photon/s·mm^2^. The median X-ray beam energy passing through a 1 mm-thick silicon wafer was 24.5 keV. The X-ray beam size was 8 mm (H) × 5 mm (V). The test section was placed approximately 30 m downstream from the X-ray source. A CsI scintillator with 500 μm thickness was utilized as the scintillator crystal. The distance between the scintillator and a test sample, which is an important parameter in phase contrast X-ray images, was fixed at 53 cm. X-ray images were consecutively recorded with a high-speed camera (SA 1.1, Photron, Japan). The field of view was 1945 μm × 1945 μm (1024 × 1024 pixels) at 10× magnification.

X-ray images were recorded at 500 frames per second (fps) for 2 s. The two-frame cross-correlation PIV algorithm was applied to obtain velocity field information of the blood flows. The interrogation window size was 64 (horizontal) × 32 (vertical) pixels with 50% overlap. The size of the interrogation window is different from that of WSS measurement near the vessel wall. X-ray images contain information on all particles positioned inside the X-ray beam propagation. Therefore, a mathematical formula and the deconvolution of peak smearing technique are usually applied to obtain velocity field information in the center region of the pipe flow^3^,^13^,^41^. Considering the flow and geometric features in the stenosed vessels, the deconvolution of peak smearing technique was applied in this study. The principle and usage of this technique have been demonstrated by other research groups^8^,^41^. These analytic methods were employed to analyze the experimental data obtained in this study.

### WSS measurement

WSS is the product of dynamic viscosity and shear rate on a vessel wall. The dynamic viscosity of the blood tested in this study was 3 × 10^−3^ kg/m·s. PIV analyses were conducted by focusing on the vessel wall regions to calculate the shear rate in the near wall region. Figure 6b shows a schematic of velocity vector measurement in the near wall region. First, the section near the vessel walls was demarcated from the captured X-ray images. The edges of the vessel walls were selected by adopting the threshold method; the threshold values were determined based on the standard deviations of each image. Second, the normal vector (n_1_) was obtained at each pixel located on the edge of the vessel walls. The corresponding interrogation window (s_1_) was rotated by *θ* degree defined as the angle between the vectors n_1_ and n_1_’ (perpendicular to the X-coordinate). Thus, all of the interrogation windows along the edge of a vessel wall are aligned in a single line. The size of the interrogation window near the vessel wall was 32 (horizontal) × 16 (vertical) pixels with 50% overlap. This rectangular-shaped interrogation window is effective in PIV analysis of near-wall flows because the displacement of tracer particles in the near wall region is much smaller than that in the center regions.

## Additional Information

**How to cite this article**: Park, H. *et al*. *In vivo* measurement of hemodynamic information in stenosed rat blood vessels using X-ray PIV. *Sci. Rep.* **6**, 37985; doi: 10.1038/srep37985 (2016).

**Publisher's note:** Springer Nature remains neutral with regard to jurisdictional claims in published maps and institutional affiliations.

## Supplementary Material

Supplementary Information

## Figures and Tables

**Table 1:** Geometrical parameters of three stenosis clips tested in this study.

Severity S	26%	36%	54%
Inner diameter of the vessel D (mm)	1.04	0.96	1.05
Radius of the stenosis throat *r*_*0*_ (mm)	0.77	0.614	0.484
Length of the stenosis L (mm)	1.92 (1.85 D)	1.66 (1.73 D)	1.54 (1.47 D)
Severity of the clip S_c_	30%	40%	50%
