# Supplementary material for: In vivo measurement of hemodynamic information in stenosed rat blood vessels using X-ray PIV
Source: Sci Rep. 2016 Nov 28;6:37985. doi: 10.1038/srep37985 (PMC5125094; doi:10.1038/srep37985)
Supplement: Supplementary Information [file srep37985-s1.pdf]

Scientific Reports

Supplementary information

In vivo measurement of hemodynamic information in stenosed rat blood vessels using X-ray PIV

Hanwook Park[a], Jun Hong Park[a], and Sang Joon Lee[a,*]

[a] Center for Biofluid and Biomimic Research, Department of Mechanical Engineering, Pohang University of Science and Technology (POSTECH), Pohang, 790-784, South Korea.

* Corresponding author.

Tel.: +82 54 279 2169; Fax: +82 54 279 3199.

E-mail address: sjlee@postech.ac.kr (S.J. Lee)

Supplementary

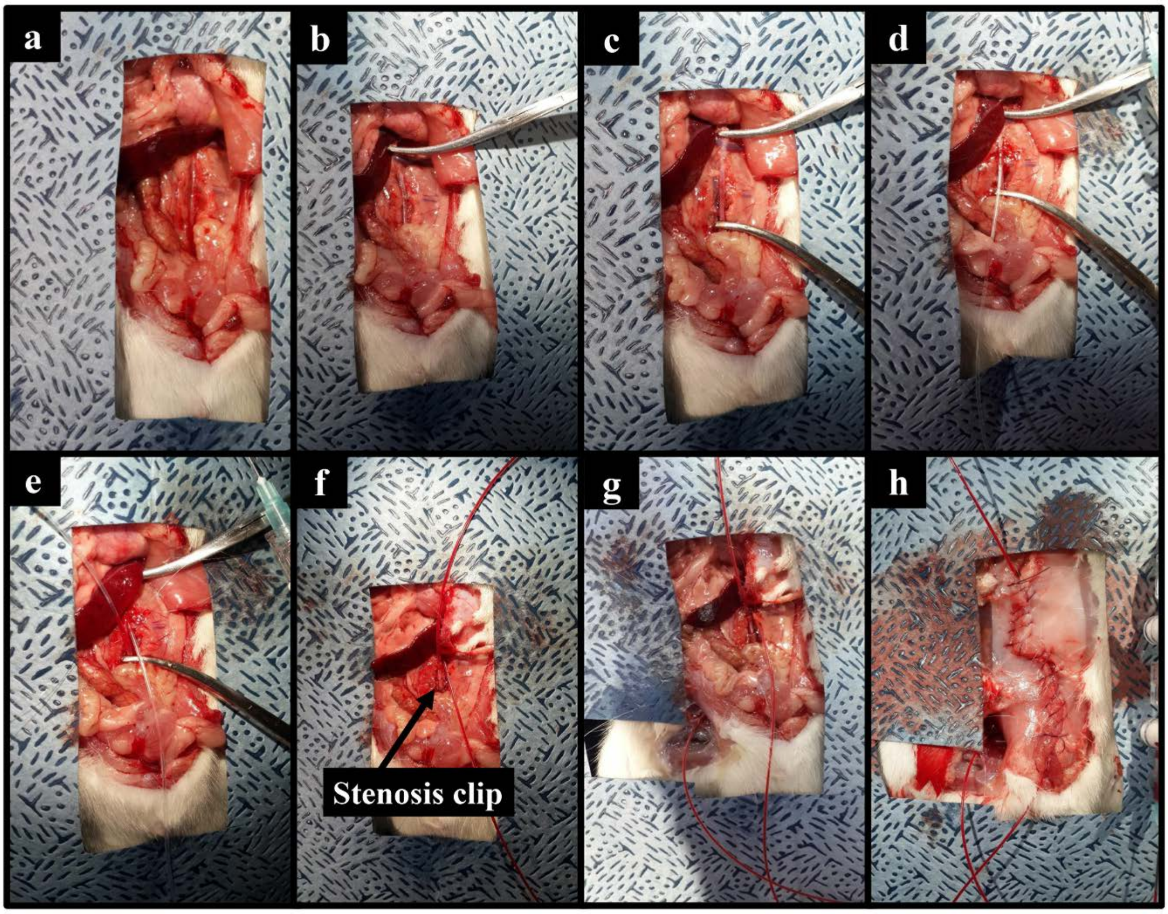

Figure S1. Surgical procedures to establish an *in vivo* rat stenosis model.
